# A political economy analysis of the legislative response to unhealthy food and beverage marketing in Chile, Canada and the UK

**DOI:** 10.1186/s12992-024-01093-1

**Published:** 2025-02-20

**Authors:** Fiona Sing, Sally Mackay, Boyd Swinburn, Kelly Garton

**Affiliations:** School of Population Health, University of Auckland, Auckland, 1023 New Zealand

## Abstract

United Nations bodies call for legal responses to restrict children’s exposure to unhealthy food marketing; however, few governments have introduced legislative controls. This research studies the underlying political economy influences that impacted the introduction of legal responses. We used a multiple case study methodology to examine the political economy influences on the policy process in three contexts (Chile, Canada and the UK). Data from documentary evidence and 21 semi-structured key informant interviews were analysed using a political economy framework studying the institutions, interests, ideas and the associated power dynamics that shaped the policy process. The prevailing neoliberal ideologies and overarching institutional paradigm, in which all actors were operating, meant any form of government intervention had to be justified, evidence-based and no more intrusive on commercial enterprise and public life than necessary. The neoliberal paradigm permeated each of the political economy elements (institutions, ideas and interests). In addition, its influence was observed in all stages of the policy process, from introduction through to adoption of the resulting law or regulation, and experienced in both the executive and legislative branches of government. A paradigm shift away from the protection and primacy of commercial enterprise and limited government interference would reduce the barriers governments face when introducing legislative responses to unhealthy food marketing. These dynamics may be tempered if institutional, actor and discursive power is harnessed in support of the legitimate public health measure, which would involve a strong mandate for the ministry responsible and a dedicated and influential policy entrepreneur.

- Provides an insight into how three different governments legislated unhealthy food marketing.

- Provides lessons for other governments about what challenges those countries faced and how they overcame them.

- Considers the political reality behind policy making that impacts on how evidence-based policy making occurs.

## Introduction

Overweight and obesity, as well as multiple non-communicable diseases including cancer, diabetes, heart disease, stroke, mental and dental health issues are associated with dietary patterns that include unhealthy and ultra-processed foods [2]. An established body of evidence shows that marketing impacts children’s preferences, requests, nutrition knowledge and dietary intake [8, 26, 31, 49, 50]. One influential driver of these dietary patterns is the insidious and prolific marketing techniques used by food and beverage companies to drive sales and profit [30].

Government action to restrict such marketing practices that children are exposed to has been on the global health agenda for two decades. Two United Nations agencies, the World Health Organization and UNICEF advocate for immediate government action and multiple global health legal instruments have called for government action [21, 68, 72–76]. However, few governments have successfully passed a comprehensive law [48, 57, 69, 71]. This is because it is perceived to be difficult to introduce marketing restrictions for political reasons and because the design of the restrictions are perceived as technically difficult [10, 40, 41, 46].

Our study is grounded in political economy analysis, which is an analytical approach that allows for a deeper understanding of the underlying historical and institutional context in which policy decisions are made, shedding light on how actors, networks, institutions and interests factor into the realities of policymaking [23]. It also incorporates theories of power in policymaking [22, 34, 39]. Specifically, this study is concerned with the ideas and paradigms that permeate policy decision-making; the institutions, structures, norms and ‘rules of the game’ set up to reinforce these ideas; and the stakeholder interests and power that affected the policy process and outcomes [23, 45]. This research aims to understand how the underlying political economy influenced the introduction of three different laws in three case countries. In exploring how the commonalities and differences in the relevant institutions, ideas and interests (and associated power dimensions) shaped the policy process in each case, we aimed to distil key lessons for other governments wanting to introduce similar legislation.

In this paper, we examine challenges faced by the governments of Canada, the UK and Chile when seeking to introduce legislation to restrict the exposure of children to unhealthy food and beverage marketing. The cases explore the Canadian Child Health Protection Bill (Bill-S228) [11]; the UK’s restrictions on online, broadcast marketing and retail settings [58, 66]; and the Chilean Food and Advertising Law [36] and the accompanying Decrees. The technical details of each country’s regulatory approach have been outlined in a separate paper [54]. 

## Methods

The study investigated the political economy dynamics at play as government actors developed the policy and attempted to pass the corresponding legislation or regulation through the legislative branch of government.

The analysis was informed by Pettit and Mejía Acosta’s [45] approach to bridging political economy and power analysis [45]. We incorporated theory regarding the relevant influences on decision-making in relation to institutions, interests and ideas [23] and associated forms of power from Lukes [34] and Gaventa [22].

### Case studies

A case study methodology was used to explore the three experiences in each country [35]. The rationale and search strategy for the case studies are included in a separate paper [54]. However, in summary, the countries were chosen from a scan of global policy databases and the global literature base and were selected if they met the inclusion criteria that a mandatory legislative approach was adopted that restricted marketing on three or more media or settings [48, 57, 69–71, 75]. Other considerations included adequate access to appropriate documentation and key informants.

The cases were time-bound starting from the introduction of the policy issue on the political agenda through to the development of the government policy document and subsequent draft legislation and supporting regulations that outlined the technical details of implementing the law (where applicable). Table 1 provides an outline of the time parameters and key milestones of the cases in question. In the case of the UK, the study was time bound to the passing of the law and subsequent pause of the implementation of the law in May 2022.

**Table 1:**
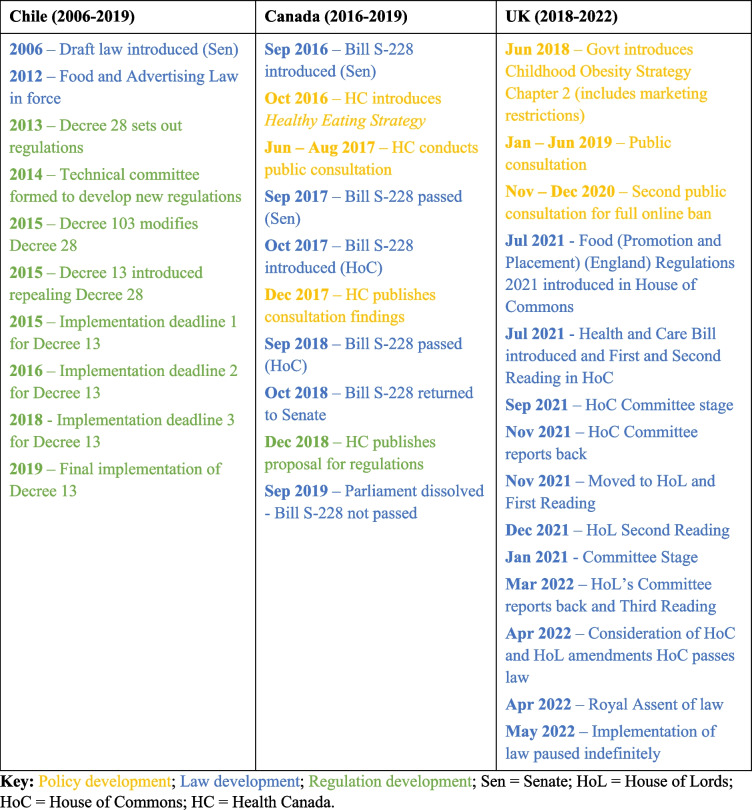
Timeline of key milestone events in policy process

### Data collection

Details of the data collection methods have been explained in detail in a separate paper [54]. Publicly available documentation from government sources was collated along with peer-reviewed literature and grey literature. Further, 21 semi-structured key informant interviews from Chile (*n* = 3), Canada (*n* = 10) and the UK (*n* = 8) were undertaken to garner further information. Key informants included policymakers and politicians, academics and civil society advocates who had close knowledge of the policy process of the three cases because they worked directly on the policy reform.

### Data analysis

To analyse the data, we developed a conceptual framework based on the political economy analysis literature that utilised the 3Is: institutions, interests and ideas [9, 23, 61, 63]. Informed by the political science literature on power, the authors also included three different types of power associated to each of the 3Is in the conceptual framework [22, 34, 37]. To further strengthen the conceptual framework, other policy process theory literature that dealt with similar aspects to the 3Is and their associated power dynamics were also considered and synthesised into the framework [4, 7, 9, 19, 22, 24, 27–29, 34, 37–39, 51, 52, 63]. All authors then analysed and amended the conceptual framework to reach consensus and this framework formed the basis of the coding schema. Table 2 outlines the agreed explanation of each key concept integrated into the conceptual framework.

**Table 2:** Conceptual framework of political economy elements

Concept	Explanation	Associated power dynamic
Institutions	‘Institutions’ refer to two different things: the formal institutions (for example organisations such as the WHO or institutions like government agencies); or the rules, structures, norms, authority and values of such an organisation or institution that structure or influence the way it makes decisions or acts [7, 51, 52, 63].	**Institutional or structural power**: Power that allows certain actors to shape, or engage in, rules and institutional practices/arrangements or make the rules themselves. This type of power can be drawn from underlying economic and institutional structures and processes that place certain actors in positions of power [22, 34, 37].
Interests	Stakeholder ‘interests’ refer to the objectives and goals of different actors, and what they see as important [7, 51, 52, 63].	**Actor power**: refers to which actors directly influence policy and decision making, and mechanisms through which this occurs [34, 52, 61]. Also known as instrumental power.
Ideas	‘Ideas’ refer to how policy actors understand or frame an issue, shaped by their values, beliefs and ideologies. It includes not just the actors’ perceptions but also concepts and theories communicated by the actors in the discourse around the policy process [7, 51, 52, 63].	**Discursive power**: The power to shape and influence the policy process through discourse and language. It is a power exercised through different vehicles, i.e., the media or political lobbying or debate. It can focus on the framing of the policy issue, or the actors involved; or utilise the influence of broader norms (i.e., political or societal) [19]. Also known as ideological power.

A case study summary was constructed for each case informed by the document review and interviews. After agreeing on the conceptual framework and reviewing the case study summaries, the authors agreed on a coding framework informed by the conceptual framework.

FS undertook iterative rounds of coding of each case. KG reviewed the updated coding summaries and the authors conferred on any differences and agreed on final data coding. From there, FS carried out a secondary analysis of the overarching themes present for each case to determine the overarching political economy factors present across all cases. The research team reviewed and discussed the preliminary findings at each stage of analysis.

## Results

The political economy elements were studied in two branches of government: the executive branch (the ministry level and the ministerial level) and the legislative branch (the law-making parliamentary level). However, the policy processes were different in each of the three cases. For example, in Chile, a Senator introduced the law into the Senate where it was eventually passed (legislative level), then the regulatory development was given to the Ministry of Health to lead (executive level). In Canada, the draft legislation and the policy development happened concurrently as a Senator introduced the Bill at the same time the Government decided to pursue the policy. However, in the UK the law was a government bill sponsored by the relevant government department at the executive level and the Secretary of State for Health at the legislative level. Therefore, the political economy factors differed for each case as the power dynamics changed and the actors involved varied.

However, the analysis showed that the institutional norms and the corresponding power dynamics dictated how the relevant actors and their interests were positioned and resourced with respect to actor power, and how ideas and discursive power were applied.

### Institutions

Prevailing neoliberal ideologies and institutional norms, even in three different political contexts, had the most profound influence on the policy process. The overarching institutional norm, in which all actors were operating, was that any form of government intervention must be justified, "evidence-based" and no more intrusive than necessary on commercial enterprise and the lives of the population. This can be seen in all stages of the policy process from the introduction of the policy through to the adoption of the resulting law and was experienced at both the executive and legislative branches of government.

To have the best chance of surviving the policy process and successfully being passed into law, policies needed to be considered a cost-effective and proportionate response to the problem attempting to be addressed. The impact on the economic welfare of companies or the national economy were of utmost concern, and all actors operated within that paradigm, navigating those dominant concerns. This institutional feature is evident in public consultation submissions, public discourse and parliamentary debates in relation to the impact that any marketing restriction would have on the ability for companies to trade, both domestically and internationally.

#### Executive branch of government

The Ministries of Health in all three cases established the policy parameters carefully to navigate these institutional norms. They buttressed their policy scope with scientific evidence to prove the necessity for the policy and to justify its design, and then the subsequent law or regulations. To increase the legitimacy of the policy scope, they used external actors to support the policy design: either academic experts, civil society groups, or both. In Chile and Canada, they used the support of UN level actors, such as the WHO or PAHO, to add legitimacy to the policy. They worked with government lawyers to meticulously assess the risk of the policy scope, delicately navigating the fine line between a comprehensive far-reaching policy with optimal potential effectiveness with a policy that could withstand challenge from various actors in opposition. Most proponents of the law expressed it was important that some legal instrument was passed rather than preserving the best practice design based on the evidence available.

In all cases, there were public health actors who did argue for a wider scope of the proposed policy. However, in Canada the Ministry of Health worked closely with Stop Marketing to Kids, a civil society group representing a united front of multiple organisations, to garner support. Government key informants expressed how critical working with these umbrella groups was to the success of the policy navigating the policy process and other opposing actors.

In Chile and Canada, the executive branch of government (the Ministers) had to unanimously support the regulations (decrees) (Chile) or the policy (Canada) in order to get presidential or prime ministerial sign off to proceed. This required further debate with executive branch actors who had different interests to protect. For example, those in support of the measure and those actors in ministries that supported the food and beverage or advertising industry who wanted reduced regulatory intervention. The Ministries of Finance or Agriculture required the most persuading and Ministry of Health policymakers and senior officials negotiated the policy with those actors. In the UK, the then Prime Minister, Boris Johnson, removed this step of the process by incorporating it straight into a bill going before the Houses of Parliament.

The executive branches of government were also responsible for formally consulting widely with the public and incorporating any appropriately supported amendments received from those consultations. The degree to which the ministries responsible engaged with all actors either in support or opposition to the policy depended on the constitutional structures in place. In Canada, senior leadership introduced rules disallowing contact between industry actors and the policy team. In Chile, policymakers met with industry actors about their concerns with the policy. The process around formal or informal meetings with industry actors in the UK was less clear.

#### Legislative branch of government

After the policy scope was debated in the executive branch of government, the overarching law that would implement that policy was subject to debate in the legislative houses of parliament. In all cases, there was a two-house system, consisting of an upper house (Senate or House of Lords) and a lower house (House of Commons). This meant that the law faced scrutiny by actors in both houses, sometimes multiple times. Parliamentarians who opposed the law challenged the introduction of the law or attempted to introduce amendments to the scope of the law. In Canada and the UK, the laws also faced intense scrutiny at the committee stage, where a select group of parliamentarians called expert witnesses who opposed or supported the law to defend their positions about the necessity or scope of the law. Public health actors, such as academics or civil society organisational representatives and food and beverage industry actors were questioned by the parliamentarians.

Throughout this policy process, the food and beverage industry were engaged in various types of corporate political activity. This involved directly approaching decision makers, either in the ministries of health or other more sympathetic ministries such as finance or agriculture; hiring public relations firms to help sway public opinion through influencing the discourse on the policy in the media; or working through parliamentarians in either of the two houses to garner support for abandoning the regulations or significantly weakening their effect.

#### Institutional power

The neoliberal paradigm underpinned the institutional norms and structural power dynamics, with the food and beverage industry and the advertising industry wielding substantial power because of the value attributed to their contribution to the national economy. However, in all three cases there were strong mandates to act to introduce legislative responses to the issue, albeit from vastly different sources, that increased the legitimacy of the public health cause. In Canada, the Prime Minister included marketing restrictions in his public mandate letter to the Minister of Health providing a strong mandate for Health Canada to act on this issue.

Senator Greene Raine, who was championing the Bill, also had considerable influence in the Senate, particularly in her Conservative Party, but also across party lines, as a much-respected former olympian. Obesity policy had been on the UK Conservative Party’s political agenda for over a decade, and various prime ministers had considered the issue of marketing restrictions. However, the inclusion of mandatory restrictions in Boris Johnson’s Government’s Childhood Obesity Strategy meant that the Department of Health and Social Care could pursue this policy option.

In Chile, Senator Guido Girardi championed a comprehensive law regulating food labelling, school settings and advertising that passed in 2006 and the role of implementing the law was given to the Ministry of Health. Therefore, the law and the decree development was not a government-sponsored law, so while the Ministry was involved in the Decree development, the Government in power was not responsible overall for the policy response. Despite that, the legislation being in place meant that there was a strong mandate for the Ministry of Health to act in this area. The Ministry was also trusted at this time with nutrition issues, as they had successfully combatted undernutrition a decade earlier. The Ministry of Health convened two expert groups of academics to work on the details of the law which they perceived added legitimacy to the policy design, as it was based on expert evidence.

### Interests

Broadly speaking, the actors could be described as proponents or opponents of the policy. Those opposed to the law were concerned that the government was overreaching into the lives of the public and that it would negatively impact the economic position of companies in which they had an interest, or the national economy. These actors included the food and beverage industry, including their representative groups (such as AB Chile or the Canadian Beverage Association), politicians who were ideologically aligned with economic growth or whose portfolio was responsible for regulated industries, or parliamentarians in opposing parties to those bringing in the law.

Proponents of the policy considered policy action was necessary to improve or protect human health. In the three cases these included: Senators Girardi (Chile) and Greene Raine (Canada) who were the political champions who introduced the Bills; the Ministries of Health including civil servants, policymakers and senior officials; academics whose research supported the policy; and civil society groups, usually aligned on health issues like Non-communicable disease (NCD) prevention.

#### Actor power

Canadian Conservative Senator Greene Raine shepherded her Bill through the policy process, negotiating across party lines to achieve non-partisan support for the Bill. However, Senator Greene Raine was required to retire because of age thresholds before her Bill could reach the final assent. In the final stages of the Bill a select group of Senators held up the Bill from receiving final assent before the Senate disbanded before an election in a political move called ‘filibustering’. An information leak uncovered that representatives from major multi-national food and beverage companies had written to the Senators who eventually delayed the Bill, asking for their support with stopping the Bill coming into force [16].

Chilean Senator Guido Girardi was a policy entrepreneur for the Food and Advertising Law, in partnership with a revered Chilean academic, Professor Ricardo Uauy. In contrast, the then UK Prime Minister, Boris Johnson, acted as haphazard policy entrepreneur for the UK policy, after allowing the policy to return to the political agenda in 2020 and then fast tracking the policy through the policy process. Once the legislation was passed, he then retracted his support for the policy by stalling its implementation, bowing to the pressure of those inside his caucus who were opposed to the policy due to the potential effects it could have on the food and beverage industry. The retail price and promotion restrictions were called into question in a cost-of-living crisis, and this argument was used to stop the entire legislative framework – including the broadcast watershed and online ban, although unrelated to the cost-of-living issue.

At the executive level of government, policymakers interviewed from Canada and Chile expressed their confidence that they were not required by senior management in the ministries to incorporate all of the amendments argued for by opponents to the policy. This came from an overarching sense of mandate. Canadian civil society actors also communicated that Health Canada demonstrated strength in maintaining their ground on a strong policy approach.

### Ideas

The findings in relation to the ideas or framings used by various actors in the three cases, whether opponents or proponents of the policy, aligned with the institutional norms in which the actors were operating.

For example, proponents of the policy focused on communicating to other actors the necessity of the policy to meet a legitimate health objective by outlining the size of the obesity problem, the amount of marketing children are exposed to, and the need to protect children from such harmful marketing practices. Canada used a health protection framing rather than a health promotion framing, due to the legislative framework chosen to introduce the law change – an amendment to the Food and Drug Act, which is a criminal act to protect health. This meant the policy was aimed at protecting the population from ill health, with a focus on vulnerable populations, not promoting healthy choices. In contrast, the UK’s Childhood Obesity Strategy focused on creating enabling environments that allowed the population to make healthier choices.

Chile and Canada focused on a child health framing, while the UK’s policy was ultimately designed to protect the whole population from unhealthy food marketing, even though the mandate for the policy came directly from the Conservative Government’s Childhood Obesity Strategy. Key informants felt the UK Government seized the opportunity given by the Covid19 pandemic to move away from solely protecting children with the policy because many adults with obesity suffered poorer outcomes from Covid19 infections [55, 67]. This was triggered by Boris Johnson publicly stating his Covid19 illness was more serious because of his weight [14].

All government proponent actors used the framing that not acting to curb childhood obesity would have a negative effect on the nation’s economy in the long-term. In all three cases, the government articulated that a policy response that addressed the wider food environment in which children were living, as opposed to an individualistic approach like educating children about unhealthy food, was required. This required a decisive shift away from the prominent ideological framing of individual responsibility to that of systemic change and in some cases to the social determinants of health. However, in the UK, the Government also focused its framing on ‘giving people back their free choice’ and ‘helping people do what they are already trying to do with weight management’ [65].

The Chilean Government proponents adopted a framing that explained the marketing policy as a structural change that was part of a wider package of policies, all introduced under the Food and Advertising Law. While the UK and Canada didn’t have this as a dominant framing, their marketing policies were part of a wider suite of complementary policy options the respective health ministries were developing.

A child rights-based framing, or human rights framing, was not used in any of the cases and the legal obligations of the government under the United Nations Convention on the Rights of the Child (UNCRC) were not raised in the discourse [13]. Chile did have a policy objective to protect children, but the discourse did not use a rights-based approach. Key informants explained that because the mandate for the policy sat with the governing health agency and relevant ministers, who did not have an express child rights mandate, it was harder to use this framing. There was also some confusion expressed as to how a child rights-based framing could be utilised, although many key informants agreed it would have been a helpful framing to use.

In response, actors opposed to the policy expressed concern at the potential economic impact. Common framings from actors opposed to the policy included that there would be: detrimental economic impact on companies and the national economy; unintended consequences; or a negative impact on small businesses or well-loved national brands. Further framings focused on the narratives that: protecting children is the responsibility of parents; there was a lack of evidence to support the necessity of a legislative response; the costs to businesses far outweighed the cost of NCDs; the policy restricted trade more than necessary (citing World Trade Organization Treaty implications); there were less trade restrictive ways to address the issue of obesity; commercial rights would be unlawfully impinged on; there was limited precedent in other countries to show effectiveness of the policy; and that self-regulatory systems were sufficient.

For example, in Chile, research found that 32.5% of the food industry discourse in the media before the law was introduced related to the economic threat the law caused, followed by an argument that it was too ambiguous to implement (8%) [15].

#### Discursive power

While the framing propagated by the opponents to the policy did affect the level of scrutiny of the policies and the extended length of the political process, ultimately all three laws either passed or got very close to passing. Therefore, the health framings did assist to persuade the actors involved of the necessity for the policy in that they achieved executive government assent, and proceeded through both Houses of Parliament.

In Chile, the framing of a structural response to the large obesity problem was successful in getting the law passed and decrees developed, albeit over a period of nine years from the Bill being introduced in the Senate until the final Decree passed. In contrast, the opponents’ framings, as well as the institutional norms and ideological paradigms under which the actors were working, did result in the delay of the implementation of the UK law and the Canadian Bill from being passed. 

## Discussion

### Summary of findings

This research explored how the underlying political economy influenced the introduction of three different laws in three case countries. It analysed how the commonalities and differences in the relevant institutions, ideas and interests (and associated power dimensions) shaped the policy process in each case, so that other governments can learn from those experiences.

Ultimately, the prevailing neoliberal ideologies and institutional norms, in three different political contexts, had the most profound influence on the policy process. It took an extraordinary alignment of forces, notably including a strong, long-term policy entrepreneur from inside government, and a dominant Ministry of Health with dedicated civil servants to push the policy through the process with a strong mandate to act in all three cases. The political economy elements present in the institutions, interests and ideas and associated types of power conspired to prevent passage of the Canadian Bill S228.

### Implications for public health

Analysis across all three cases highlighted that, for a policy to survive the policy process, pass into law and be implemented, there needs to be sufficient alignment of institutional/structural and discursive power by the proponents of the legislative response. This alignment may enable the prevailing constraining institutional norms to be tempered to some extent.

We observed four elements that can increase the policy’s chances of success. First, a committed and influential policy entrepreneur, in Parliament, is needed, particularly to shepherd the law through the legislative process. This is in line with Kingdon’s work on the importance of policy entrepreneurs in influencing policy change [28, 29]. Depending on the type of bill this can be a parliamentarian in, or outside, of the government. In the UK, the Government took the Bill through the legislative house, and while the Prime Minister played a haphazard role of ensuring the policy did get on the legislative agenda, he was then absent for the assent through the Houses of Parliament leaving the defense of the Bill to the Secretary of State for Health and the Minister of Health. Therefore, arguably a government bill without a strong policy entrepreneur, like Senator Girardi and Senator Greene Raine, could still have a chance of success.

Second, a committed and dedicated ministry of health that has both senior leadership and civil servants invested in the strongest policy outcome is critical. This will ensure that when a policy window opens, the strongest evidence-based policy is ready to progress, and also increases the chance of the policy scope being protected through the negotiations of the policy process. This accords with other studies showing the importance of committed policymakers in championing policies through the policy process [44, 61, 62].

Third, a strong mandate must be given to that ministry of health to increase their legitimacy internally within government. Ideally this mandate would come from the prime minister or equivalent and be communicated publicly, as seen with the Trudeau mandate letters in Canada or the Childhood Obesity Strategy in the UK, and President Bachelet’s support to implement more robust decrees in Chile. This can help to level the playing field between the opponents and proponents of the policy response and shape the discourse around the policy development and legislative process. This internal legitimacy of the health sector can be an important means to bolster its institutional, actor and discursive power and reduce the policy incoherence between competing health and economic objectives [3].

Fourth, a coordinated civil society group (using discursive power) which supports the ministry of health to defend the form and substance of the policy and subsequent laws can help temper the structural and institutional power imbalances at play. According to Friel et al. [18], these groups represent one of the ‘weapons of the structurally weak’ in public health governance; public interest actors can do this in closed and claimed spaces, building on their expert and moral power, and using different frames to appeal to ‘unusual bedfellows’ [18]. Likewise, the ‘network’ and ‘moral power’ of key public interest actors can be important enablers for use of existing institutional processes and the creation of new processes [18]. Townsend et al. [64] discuss the ‘inside and outside’ strategies used by non-governmental organisations to influence policy to combat the commercial determinants of health [64]. Other studies have also found that civil society advocates are critical to support the policy process [44, 52].

However, while these elements can help temper the prevailing neoliberal ideology, a paradigm shift is ultimately required to increase the legitimacy of policy interventions that aim to protect children from unhealthy food and beverage marketing to address the commercial determinants of health. This has been repeatedly articulated in the literature examining policy inertia [33, 44, 56]. Moon [39] observed that global governance systems exhibit features of complex adaptive systems, which are difficult to change [39]. The most powerful lever of any systems transformation is a shift at the paradigm level. Abson et al. and Friel et al. envision ways to create the necessary structural shift to change the dominant narrative from individual responsibility to state and commercial responsibility, prioritise people (and the environment) over profits, and reaffirm social and political rights and norms in the public interest. Structurally less powerful actors can (i) narrate a different possibility, (ii) work in coalitions to articulate and lobby for structural reforms, and (iii) drive institutional change through engagement with institutional processes [1, 17].

The case studies show that a focus on the health issue and the need for upstream structural responses was helpful but ultimately not effective enough on its own to support the policy through to implementation. Utilising the child rights-based framing and increased legitimacy provided to governments because of their duty to fulfil their obligations under the UNCRC is an important area that requires further knowledge translation and ultimately capacity building [20, 25, 53, 75].

### Contribution to the literature

This paper adds to previous research that investigated the political economy of NCD prevention policy in seven sub-Saharan countries [61] and Thailand [46], and in particular food marketing restrictions in Fiji [62], Thailand [47], Brazil [10] Malaysia [40], and Australia [43]. These studies similarly found that industry actors influenced the policy process and similar institutional challenges created barriers for policymakers [10, 40, 46, 61, 62]. For example, Thow et al. found that the ‘entrenched economic policy paradigms’ favouring industry-led economic growth and strong industry stakeholder opposition caused challenges for the introduction of beverage taxes in the sub-Saharan countries studied [61]. In the food marketing policy studies, Malaysia and Australia displayed strong institutional rule-making bias toward self-regulation and voluntary approaches, and Brazil’s policy environment was permeated by corporate political activity and commercial influence through ‘informal governance’. Policy incoherence within government, wherein regulation of business activity via marketing restrictions contradicted more dominant economic policy goals, as well as power imbalances between industry and public health stakeholders in policymaking were also found in the case study of Fiji [62]. Yet, in each of these studies, regulatory marketing restrictions either had not been passed [10, 40, 43, 47] or had passed but were not well implemented nor as comprehensive as the regulations presented in this case study [47]. This paper extends the literature as it examines three cases of relative success, representing some of the most comprehensive marketing restriction regulations attempted to date. It distills the legislative experiences from three distinct countries – geographically, economically, and socially – for policies that have been passed and implemented (Chile and UK imminently) and one that failed to pass yet got very close (Canada). By focusing on the details of the legislative process, this study provides the best available guidance to a policymaker audience willing to attempt comprehensive regulation of unhealthy food and beverage marketing. This paper may be particularly helpful if considered alongside Ngqangashe et al’s [43] examination of the inhibitors and supporters of unhealthy food and beverage marketing regulation in Australia, which focused on the regulatory, normative and cultural cognitive pillars within existing institutions [43].

Comparing three cases and the political economy dimensions and power attributes has found that despite the differences in the case country and the policy outcome, there are similar dynamics at play that can influence the success of failure of a policy process. These findings are also consistent with the wider political science literature, particularly those that have found the policy entrepreneurs [28, 29], committed policymakers [44, 62] and civil society advocates are critical [44, 52]. This study provides a case study example of how actors receive legitimacy and exercise power contributing to the literature base in this NCD policy [3, 18, 41, 42, 63] and how the neoliberal paradigm is influencing the policy process [33].

### Strengths and limitations

The strength of this research is the use of the case study methodology to provide a deeper assessment across three cases, using a wealth and depth of data collected from multiple sources coupled with the policy process theory. An analysis using political economy theory, can provide policymakers with a better understanding of the political economy factors in other countries [5, 6, 12, 32, 59, 60]. This approach can complement policy evaluation studies, that measure how much an implemented policy has reduced children’s exposure to unhealthy food marketing, as policy process theory can illuminate key political dynamics and actor power imbalances to further support policymakers through the policy process [59, 60].

The small number of interviews for Chile is a limitation of the study, however this was alleviated by the ample amount of published material on this case and access to key experts who were particularly knowledgeable and intimately involved with the design process. There was less literature and government documents available for the Canadian case study, therefore more interviews were required with a broader range of key informants. Due to the proximal timing of the legislative process of the UK law and the research period, only a few key informants were available as most government officials could not speak about a Bill going through the Houses of Parliament. Nevertheless, because comprehensive government documents were publicly available during the policy process, including public consultation documentation, this significantly aided with the analysis. Key informants outside of Government were critical to provide context that related to the political economy of the policy process.

Finally, for ease of analysis our conceptual framework was limited to the 3 I’s and associated power dynamics, in line with previous studies [61, 63]. However, other frameworks such as Moon’s [39] typology of eight kinds of power [39], or Friel et al.’s [18] Health Equity Power Framework [18] might have allowed a more nuanced analysis of how power played out in the policy processes of these cases.

## Conclusion

A paradigm shift away from the protection and primacy of commercial enterprise and limited government interference is necessary to reduce the barriers placed on governments introducing legislative responses to unhealthy food and beverage marketing. A child rights-based framing could provide a useful avenue for a paradigm shift in terms of a legal imperative for governments to intervene. But also, if institutional/structural, actor and discursive power can be harnessed in support of the legitimate public health measure, which would involve a strong mandate for the ministry responsible and a dedicated and influential policy entrepreneur, these dynamics may be tempered.

## Data Availability

The datasets from this study are unavailble due to the confidentiality and privacy agreements made between the researchers and the study participants in line with our ethics obligations.
